# Comparison of Different Commercial Conducting Materials as Ion-to-Electron Transducer Layers in Low-Cost Selective Solid-Contact Electrodes

**DOI:** 10.3390/s20051348

**Published:** 2020-02-29

**Authors:** C. Ocaña, M. Muñoz-Correas, N. Abramova, A. Bratov

**Affiliations:** 1 BioMEMs Group, Instituto de Microelectrónica de Barcelona, Centro Nacional de Microelectrónica (IMB-CNM-CSIC), Campus UAB, Bellaterra, 08193 Barcelona, Spainmireia.minoz@imb-cnm.csic.es (M.M.-C.); natalia.abramova@imb-cnm.csic.es (N.A.); 2 Laboratory of Artificial Sensors Systems, ITMO University, Kronverskiy pr. 49, 197101 St. Petersburg, Russia

**Keywords:** pH, solid-contact, conducting polymer, potentiometry, ion-selective electrodes

## Abstract

Simple, robust, sensitive and low-cost all-solid-state ion-selective electrodes (SCISEs) are of interest in different fields, such as medicine, veterinary, water treatment, food control, environmental and pollution monitoring, security, etc. as a replacement for traditional ion-selective electrodes with liquid inner contact. In spite of their potential advantages, SCISEs remain mainly in the research laboratories. With the motivation of developing simple and low-cost SCISEs with possible commercial applications, we report a comparison study of six different commercial conducting materials, namely, polypyrrole-block-polycaprolactone (PPy-b-PCaprol), graphene/poly(3,4-ethylenedioxythiophene):poly(styrenesulfonate) (PEDOT:PSS) ink, poly(3,4-ethylenedioxythiophene):polyethylenglycol (PEDOT:PEG), high conductivity PEDOT:PSS, polyethylenimine (PEI) with PEDOT:PSS for their possible use as ion-to-electron transducer in polyurethane based pH-SCISEs. Among all studied pH-SCISES, PEDOT:PEG based electrodes exhibited the best results in terms of sensitivity, reproducibility and lifetime. Finally, these sensors were tested in different real samples showing good accuracy.

## 1. Introduction

Over the last decades, solid-contact ion selective electrodes (SCISEs) have received considerable interest due to their simplicity of use, portability, affordability, and flexibility. Specifically, nearly 200 papers have been published during the last five years [1]. The advantages of SCISEs over the conventional ion-selective electrodes (ISEs) have been demonstrated in a variety of applications, including medical analysis [2,3,4,5,6], agriculture [7,8], environmental [9,10,11] and water quality monitoring [12,13,14] and process control [15]. Improvements in signal stability, detection limits, reduction of water accumulation at the SC-membrane interphase, transmembrane ion fluxes, reproducibility, and research efforts in the development of new SC materials have been reported [14,16,17,18,19,20]. However, only few commercial analyzers utilize this type of sensors, such as an i-STAT single-use cartridge for blood electrolyte analysis [21], and Horiba Cardy single-ion analyzer for the analysis of small sample volume in diverse applications [22].

Among the large variety of the proposed solid-contact (SC) compositions used to establish a stable and reversible contact between an ion-selective membrane and metal, conducting polymers with mixed electron-ion conductivity have received the most attention [23,24,25,26,27]. Incorporation of nanostructured materials into the conducting polymer layer permits to obtain high double layer capacitance, thus enhancing potential stability of SCISEs and lowering detection limits [28,29]. However, Lindner and Gyurcsányi [30] mentioned that there is no consensus on the best SC design, which may be used as a standard for comparison in testing new SCISEs. “This could be due to the lack of clearly understanding the processes in the interphases SCs-membranes”. 

Concerning SCISEs based on conducting polymers, the most common ways to deposit the material onto the electron conductive substrate are via electrochemical or chemical oxidation. Though these methods give satisfactory results, they are not suitable for mass sensor production. Drop-casting, spin-coating and inkjet printing seem to be the best alternatives. However, many publications report that films of the same conducting polymer obtained using distinct deposition methods show different characteristics [31,32]. These factors affect the reproducibility of standard potentials and the potential stability, important characteristics of ISE for their mass production.

The objective of this work was to test different commercially available conducting polymers deposited by drop-casting, thus forming SCISEs, in order to eliminate the process of in-situ synthesis of a CP over a metal contact be electrochemical or chemical oxidation.

In the present work, a comparative study of six different commercial conducting materials, namely, polypyrrole-block-polycaprolactone (PPy-b-PCaprol.), graphene/poly(3,4-ethylenedioxythiophene):poly(styrenesulfonate) (PEDOT:PSS) ink, poly(3,4-ethylenedioxythiophene):polyethylenglycol (PEDOT:PEG), high conductivity PEDOT:PSS, polyethylenimine (PEI) and PEDOT:PSS has been tested for their possible use as an ion-to-electron transducer in pH-sensitive SCISEs with photocured polyurethane membrane. This research includes electrochemical characterization of SCISEs and the conducting materials, chronopotentiometry of SCISEs and studies of their lifetime under different conditions. Additionally, pH-SCISEs with optimized pH-SCISEs were tested in various real samples.

## 2. Material and Methods

### 2.1. Chemicals

Tetramethacrylate end-capped poly(3,4-ethylenedioxythiophene) solution (Meth-PEDOT, 0.5 wt% dispersed in nitromethane), polypyrrole-block-polycaprolactone (PPy-b-PCaprol, 0.3–0.7 wt% dispersed in nitromethane), graphene/poly(3,4-ethylenedioxythiophene):poly(styrenesulfonate) (graphene/PEDOT:PSS dispersed in DMF) ink, poly(3,4-ethylenedioxythiophene):polyethylenglycol (PEDOT:PEG, 0.7 wt% dispersed in nitromethane), high conductivity PEDOT:PSS (3.0–4.0 wt% dispersed in water), polyethylenimine (PEI, 50 wt% dispersed in water) with PEDOT:PSS (1.3 wt% dispersed in water) were obtained from Sigma Aldrich, along with KCl, K_3_[Fe(CN)_6_], K_4_[Fe(CN)_6_], KH_2_PO_4_, phosphoric acid, boric acid and acetic acid. Hydrogen Ionophore I, bis (2-ethylhexyl) sebacate (DOS), potassium tetrakis(p-chlorophenyl)borate (KTpClPB) and ETH500 were obtained from Fluka. Aliphatic urethane diacrylate (oligomer Ebecryl 270), cross-linker and hexanediol diacrylate (HDDA) were obtained from UCB. Blood gas control Level 2 test samples were purchased from Randox (London, UK). Photoinitiator 2,2- dimethoxy-2-phenylacetophenone (IRG 651) was purchased from Ciba-Geigy. All other chemicals used were analytical reagent grade and all solutions were prepared using ultrapure deionized water with the resistivity of 18.2 MΩ cm (Milli-Q system, Millipore, Billerica, MA, USA). The electrodes substrates based on thin film FR4 platform with gold leads (d = 2 mm) were supplied by LabCircuits (Barcelona, Spain).

### 2.2. Conducting Material Deposition

The main purpose of this work is to study and find a simple and practical method of fabrication of SCISEs using commercial conducting polymers suitable for their mass production. As the polymer deposition method a drop-casting technique was selected. Preparation of conducting materials and the protocols for drop-casting deposition are presented in Table S1 of the Supplementary Material section. Basically, various drops of a CP containing solution were drop-casted on the gold electrode surfaces, and the electrodes were placed on a heating plate in order to increase the evaporation rate of the solvent. 

In the case of PEDOT:PSS high conductivity film, the gold electrodes were treated by an oxygen plasma treatment for 60s in order to increase the adhesion of the polymer to the metal electrode surface. 

Taking in consideration that PEI and PEDOT:PSS are a polycation and a negatively charged zwitterion, respectively, a layer-by-layer method was employed for their deposition [32]. Firstly, a PEI anchoring layer was formed by immersing electrodes into a 1.5 mg/mL PEI solution for 20 min. After that, they were rinsed with water and immersed in 0.25 wt % PEDOT:PSS solution for 15 min. In this way a double layer PEI + PEDOT:PSS structure was formed over that metal contact.

It is important to mention that the dopant of PEDOT:PSS is noted in the polymer’s name (polystyrene sulfonate) and in the other cases the dopant was p-toluenesulfonate.

### 2.3. H^+^-Selective ISM Deposition

The photocurable ISM composition was prepared as presented previously [33]. Briefly, the main polymer composition was prepared by mixing the aliphatic urethane diacrylate oligomer Ebecryl 270 with the reactive diluent HDDA and the photoinitiator Irgacure 651 in the ratio (w/w) of 81:17:2. In the next step, 0.3 g of the main polymer composition was dissolved in 0.2 mL THF and the plasticizer DOS, ETH500, KTpClPB and H^+^-ionophore I were then added to this solution. This ISM solution was placed in an ultrasonic bath until it was homogeneous and then left unstirred for several hours to allow evaporation of THF. This resulted in the following ISM composition: main polymer mixture (61.3 wt%), DOS (37.1 wt%), H^+^-ionophore I (0.9 wt%), KTpClPB (0.2 wt%) and ETH 500 (0.5 wt%). 

Membranes were deposited by applying 5 µL of the solution with a microsyringe on the conducting solid-contact layers. After the membrane deposition the electrodes were exposed to UV light during 50 s using an UV Curing Light Lamp System PC-5000 (Dymax) with irradiance of 62 mW·cm^−2^ at a wavelength of 365 nm.

### 2.4. Cyclic Voltammetry (CV))

Cyclic voltammograms were recorded in a one-compartment three-electrode cell connected to the Autolab potentiostat equipped with a frequency response analyzer (AUT20.FRA2-AUTOLAB, Eco Chemie, B.V., The Netherlands). The conducting material modified electrode, a platinum electrode and a double junction Ag/AgCl/3 M KCl//0.1 M LiOAc served as the working (WE), auxiliary (AE) and reference electrode, respectively. CVs were recorded between −1.5 V and +1.5 V in a 10^−2^ M K_3_[Fe(CN)_6_]/K_4_[Fe(CN)_6_] at a scan rate ν = 0.01 V∙s^−1^.

### 2.5. Chronopotentiometric Measurements

Constant-current chronopotentiograms were recorded according the recommended procedure [34] in a 0.1 M phosphate buffer using the Autolab potentiostat by applying a constant current of ±1 nA to the pH-SCISEs for 60 s.

### 2.6. Potentiometric Measurements

The potentiometric response of the pH-SCISEs was measured using a 16-channel potentiometer (Lawson Labs, Inc., input impedance: 10^15^ Ω) against the double junction reference electrode Ag/AgCl/3M KCl//0.1 M LiOAc. Prior to the potentiometric sensor characterization, the pH-SCISEs were preconditioned under stirring in 0.1 M KH_2_PO_4_ buffer overnight. Calibration plots were obtained in a mixture of 0.02 M acetic, boric and phosphoric acids. The test solution pH was changed by adding droplets of 1.4 M NaOH solution and the potential readings were taken after 2 min. The exact pH values were measured using a pH-meter. In total 15 points were used for the calibrations in the pH range of 2–9.

### 2.7. Real Samples Measurements

For the pH determination in real samples, the PEDOT:PEG based pH-SCISEs were selected. Three different samples were employed: a blood gas calibration solution, a water sample from a fish farm, and water from a sauna bath. The electrodes were calibrated using a typical clinical calibration method applying two standards buffers (pH 4.01 and pH 8.00). After being calibrated, the potentiometric response of three electrodes were measured in each sample for five times and interpolated in the previous calibration plots.

## 3. Results and Discussion 

**Figure 1:**
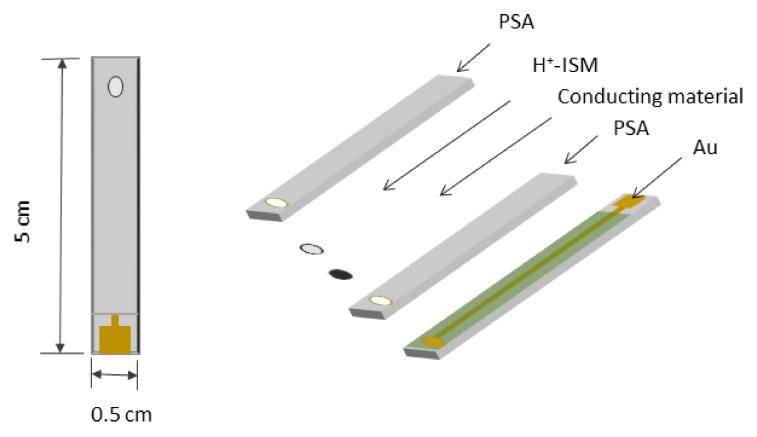
Schematic representation of the developed pH sensitive solid contact ion selective electrodes (pH- SCISEs).

Figure 1 represents the structure of the developed SCISE, which comprises a 5 mm wide FR4 strip with gold electrode, lead, and a contact pad. An ion selective membrane covers an electroactive conducting material deposited over the gold electrode, forming a solid electrical contact to the ion selective membrane. In order to isolate the conductive lead pads and to prevent water penetration, pressure sensitive adhesives (PSA) were used. 

The general operation principles of the SCISEs and essential criteria for stable potential of this kind of sensors are well known [35]. Basically, papers are focused on the development of novel SC without putting much attention to other elements of the sensor, such as the electrode substrate or the electronically conducting material. 

Despite the apparent simplicity of their design, solid contact electrodes based on conductive polymers and formed on screen printed or printed circuit boards (PCB) substrates, are rather complex structures and the properties of all the materials they are composed of should be taken into account. We can distinguish three components that are common to all electrodes of this type: the conductive polymer, the material of the electrode on which it is applied, and the insulating material, covering the substrate with an opening at the electrode and a contact pad (see Figure 1). This material is in contact with the ion-selective membrane and a good adhesion between two materials is required.

The most important is the type of the conductive polymer, its structure (bulky counter anions can suppress the pH sensitivities of polymers [4,36]) and the deposition method. Most of the studies of the properties of solid contact have been performed using classical glassy carbon (GC) disc electrode with conductive polymers obtained by electropolymerization. This method, as well as polymerization by chemical oxidation, is very inconvenient in the mass production of electrodes, especially when screen printed devices on plastic substrates are used, due to the presence of aggressive solvents and oxidizing agents required for polymerization proses. When a drop casting method of a conductive polymer deposition is used, the thickness and morphology of the obtained films will strongly depend on the polymer concentration in the drop casting solution, type of solvent, and baking temperature after polymer deposition. The properties of the obtained films may differ considerably from films obtained in another way [37]. It should also be taken into account that this method often results in inhomogeneous films, as reveal an optical image presented in Figure S1 of the Supplementary Material section. The reproducibility of such a deposition method is not high, however, according to our data it can lead to satisfactory results [23].

A more detailed study of the solid contact electrodes showed that equilibration times and drift of sensors depends not only on CP film thickness [25], but also on the type of conducting materials, type of ion selective membrane (K, Na, or pH), number of hydration/drying cycles, and type of solutions used for calibration [38]. Noble metals or materials containing various types of carbon are commonly used as a conductive material in electrodes of this type. However, in their thin film configuration, properties of these materials may differ in their bulk analogs. All these make it even more difficult to compare electrodes of different configurations when the same materials are used. In other words, the results of the study may vary from what is expected initially.

Another factor that must be taken into account is the material used as the insulating layer of the substrate with the metal electrode. As such materials pressure sensitive adhesive materials based on polytetraphthalate (PET) or different acrylic and methacrylic derivatives are most often used. As a result of our own research (data not published), it was found that the adhesion of ion-selective membranes, both based on PVC and on the basis of photopolymerizable acrylate, in the case of commercial electrode substrates (ZENSOR R&D Corp., Taiwan or Dropsens, Spain) is very low and these substrates with metal electrodes cannot be used.

Considering these factors and after testing different substrates with gold electrodes, commercial electrodes based on FR4 platform and electrolytic gold as an electronically conducting substrate (LabCircuits), have been selected.

### 3.1. CV Characterization of the Different Conducting Materials

**Figure 2:**
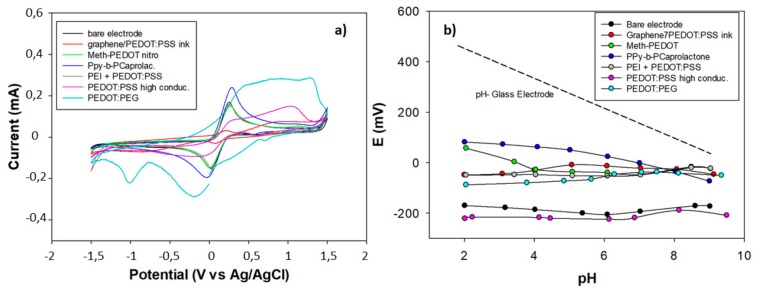
(**a**) Cyclic voltammograms of gold electrodes with different conducting polymer films. CVs were recorded between −1.5 V and +1.5 V in 10^−2^ M K_3_[Fe(CN)_6_]/K_4_[Fe(CN)_6_] at a scan rate ν = 0.01 Vs^−1^. (**b**) The potentiometric pH sensitivity of the conducting materials. The pH glass electrode response is presented with a dashed line.

Electrochemical characterization of different conducting materials films was performed by cyclic voltammetry (CV) in 10^−2^ M K_4_[Fe(CN)_6_]/K_3_[Fe(CN)_6_]. Cyclic voltammograms presented in Figure 2a show that in the presence of conductive polymers oxidation-reduction current peaks increase due to enhancement of the electric charge transfer properties of the electrodes. PPy-b-PCaprol and PEDOT:PEG modified electrodes showed much higher current under the same conditions than the others films. It is important to mention that the solutions of commercial CP materials may contain additives and impurities that could affect the electrochemical characteristics of the films, particularly the voltammogram shape, so that their properties may be quite different in comparison with electrochemically polymerized films. This is the case of the PEDOT:PEG voltammograms, where in spite of high currents, no well-defined peaks were observed. In the case of Graphene/PEDOT:PSS films and PEDOT:PSS high conductivity, unexpected decreases in the redox peaks appeared after the electrode surface modification with the polymer film. From the obtained experimental data, it may be concluded that among all studied compositions PPy-b-PCaprol and PEDOT:PEG films are the best suited materials as solid-contacts for SCISEs. 

### 3.2. pH-Response of the Conducting Materials

The influence of pH on the potential response of the different conducting films deposited on a gold metal electrode was tested in a mixture of 0.02 M acetic, boric, and phosphoric in the pH range of 2.0–9.0 (adjusted with NaOH). From the results presented in Figure 2b, it can be seen that PPy-b-PCaprol and Meth-PEDOT films showed a pH-response in the pH interval of 4–9 and 2–4, respectively. However, taking into consideration the pH response of a conventional pH glass electrode, we can consider pH-sensitivity of the other conducting films as negligible, which is beneficial for the potentiometric sensor construction since only the membrane must respond to pH. 

### 3.3. Chronopotentiometric Measurements

**Figure 3:**
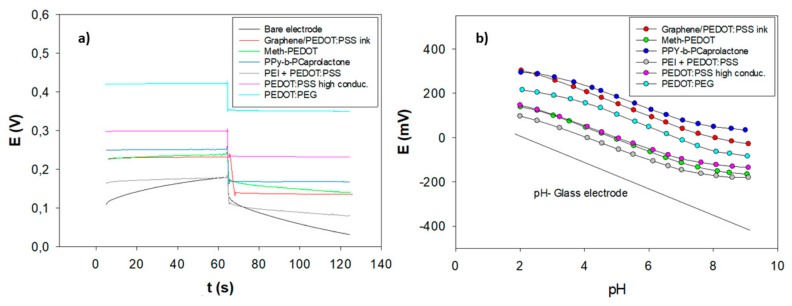
(**a**) Chronopotentiograms of the different pH-SCISEs. The measurements were done in 0.1 M phosphate buffer by first applying +1 nA for 60 s and then −1 nA for 60 s. (**b**) Calibration curves for pH-SCISEs in a mixture of 0.02 M acetic, boric, and phosphoric acids with added NaOH.

Constant-current chronopotentiometric measurements were performed recording the potential of pH-SCISEs after applying a current of ±1 nA. The slope (ΔE/Δt) of the response curves provides a direct measure of the potential stability of the pH-SCISEs, giving information on electrochemical reversibility of the interface between ion selective membrane and the metal electrode. From the results presented in Figure 3a, it follows that electrodes prepared with PEDOT:PEG and graphene/PEDOT:PSS ink SCs show the best potential stability values. From these measurements, we can also estimate the bulk resistance of the electrode (solid-contact and membrane) and redox capacitance of the solid-contact [34]. The potential jump in the chronopotentiometric curves can be employed to calculate the total resistance of the electrode, which is controlled by the bulk resistance of the polymeric membrane. As shown in Table 1, all studied pH-SCISEs had relatively high bulk resistances of 81.3–57.3 MΩ due to the inherent nature of the polyurethane membrane. 

In order to obtain a stable electrode potential in SCISEs the conducting material must show high redox capacitance, since the ion to electron transduction process constitutes an asymmetric situation due to the charge and discharge of the conducting material [34]. Assuming an acceptable potential drift of 1 mV/h and a potentiometric residual current of 1 pA in a moderately accurate measurement, it can be calculated that a minimal electrode capacitance of 3.6 µF is required [39]. The redox capacitance (C_L_) can be calculated using Equation (1) from the values of the potential drift (∆E/∆t) of the pH-SCISEs and the current (i) following the equation [31]:

∆E/∆t = C_L_/i (1)

The values of the potential drift (∆E/∆t) and C_L_ estimated by chronopotentiometry for the pH-SCISEs are shown in Table 1. The highest values of C_L_ correspond to PEDOT:PEG, PEDOT:PSS high conductivity, and PPy-b-PCaprol with 54.6 µF, 44.8 µF, and 39.2 µF, respectively. According to the results obtained by CV, Ppy-b-PCaprol shows high redox capacitance. However, this conducting polymer exhibits pH response in the pH interval of 4–9, which is not a good candidate as a SC in pH-SCISEs. Therefore, these data, along with reported above in Section 3.1 and Section 3.2, suggest that among the conducting films studied here, PEDOT:PEG films are best suited as SCs for pH-SCISEs.

It is well known that conducting polymers are photosensitive organic semiconductors [39]. Taking into consideration that UV exposure during the ISM photopolymerization may affect properties of the conducting polymer, special tests have been performed. Obtained results show that after 50 s of UV illumination of PEDOT:PEG film on the gold electrode (without membrane), no changes in the redox capacitance occur, which means that the properties of the conducting polymer remain unaltered (data not shown).

### 3.4. Potentiometric Response of the Different pH-SCISEs 

Figure 3b shows the potentiometric response of the pH-SCISEs based on the different solid-contact and Table 2 summarizes the calibrations results achieved with these pH-SCISEs. As follows from the figure and the table, all electrodes showed close to Nernstian slopes calculated from the linear part of the calibration curves. Graphene/PEDOT:PSS ink and PEDOT:PEG based pH-SCISEs showed the best sensitivity values, 54.5 mV/pH and 53.9 mV/pH, respectively. Among them, PEDOT:PEG based pH-SCISEs exhibited the best reproducibility and linear range. Although the *E*^0^ values reproducibility is not excellent, it coincides with published in literature [40] and it is quite satisfactory for a single-use device that will be calibrated before use. E^0^ reproducibility was calculated from data obtained on three different electrodes. It is important to mention that this membrane was developed for working at neutral pH (4–8) and shows deviation from Nernstian response at pH higher than 8 due to the presence of biological levels of sodium, as observed in a previous study [33].

### 3.5. Lifetime and Storage of the pH-SCISEs 

The lifetime of the different pH-SCISEs was tested using three different conditions of the sensor storage. After being calibrated for the first time, the sensors were: i) kept in 0.1 M KH_2_PO_4_ solution; ii) stored in wet conditions using a wet paper containing 0.1 M KH_2_PO_4_ solution in contact with the membrane and then were placed inside a Petri dish in order to prevent the evaporation of the solvent, and iii) stored in dry condition inside a Petri dish. Calibration of thus stored electrodes were carried out every day in order to examine how the sensitivity response of the electrodes is affected. Storage conditions are an important factor from the point of electrode marketing. If the sensors are immersed in a solution for a long time, penetration of water through the membrane may occur. Thin aqueous pools of water can be formed at the membrane/electrode interface of the sensors, resulting in potential instability. In the case of electrodes stored in a dry condition, the membrane changes its volume when it is hydrated and when it is dried. If several numbers of hydration/drying cycles are performed, the membrane may suffer mechanical instability peeling off the substrate. Storing the electrodes in wet conditions allow solving the inconveniences mentioned above, since there is no diffusion process of the membrane components through the solution or mechanical instability. Therefore, the most suitable storage method ought to be the latter. Among all the developed sensors stored in wet conditions (when the membrane is hydrated, but it is not in contact with solution), only Ppy-b-PCaprol based pH-SCISEs exhibited a Nernstian response after 8 days, difference in slope within the first day calibration, and the last day showed 7.7 mV/pH. Regarding the dry conditions, the same conducting polymer-based electrode (PPy-b-Caprol based pH-SCISEs) showed the best response after 9 days of being stored, with a discrepancy in slope within the first and the last day of 5.8 mV/pH. Finally, relating to store the sensor in constant contact with KH_2_PO_4_ solution, results revealed that Meth-PEDOT and PEDOT:PEG based pH-SCISEs presented response during 7 days. In these cases, the differences in slopes were 2.7 mV/pH and 4.8 mV/pH, respectively. These experiments revealed that PPy-b-PCaprol could be a suitable candidate as a SC material for pH-SCISES because it shows a good lifetime under dry and wet conditions. However, this conducting film shows pH response in the pH interval of 4–9 and it is well-known that it is sensitive to light and oxygen [39]. 

In view of the results reported above among studied polymers, PEDOT:PEG were chosen as the best SC material for pH-SCISEs. These electrodes being stored dry after manufacturing retain the Nernstian response (52.2 mV/pH) during two months of storage. The average E^0^ drift during this time, calculated as the difference between the initial and the final E^0^ value, was 1 mV per day. This implies that these kinds of electrodes show sufficient shelf-life to be used after being fabricated as single-used sensors.

### 3.6. The Analytical Application of the pH-SCISEs 

The feasibility of PEDOT:PEG based pH-SCISEs was investigated by measuring pH in three different samples: standard calibrator solution for a blood analyzer, water from a gym sauna (Duet Sports, Spain), and water from a fish farm (PEIMA, France), which was collected at a control point of an aquaponic system. The pH values of the samples were compared with the pH values obtained by a conventional glass pH-electrode. It is important to mention that the low pH of the sauna sample is due to perspiration wastes and dead skin cells produced in the vapor bath process. As can be seen from Table 3, the best accuracy of the sensors was obtained in blood calibration solution for blood analysis, following by the sauna sample. However, the accuracy of the sensor in the fish farm water sample was the worst. The composition of the sample was unknown and some organic components (feed, fertilizer, etc.) could interfere with the membrane, which can be translated into a worse sensor operation. 

## 4. Conclusions

Despite the advantages of the SCISEs over conventional ISE, they remain in the research laboratories. With the purpose to jump this barrier, a comparative study of six different commercial conducting materials for their possible use as ion-to-electron transducer for SCISEs mass fabrication was performed. Among different commercial thin film gold electrodes tested, one based on the FR4 thin film strip with electrolytic gold was chosen as a suitable substrate. The studied polymer films were characterized by cyclic voltammetry, chronopotentiometry and potentiometric measurements. CV did not provide important information to compare the properties of the obtained films with the in-situ electrochemically polymerized materials. The reason is the unknown composition of the commercial conducting solutions, which can contain additives and impurities affecting redox properties of the resulting polymer films. An important parameter of the SC material is the redox capacitance determined from the chronopotentiometric experiments. The highest values were obtained for PEDOT:PEG, PEDOT:PSS and PPy-b-PCaprol, 54.6 µF, 44.8 µF, and 39.2 µF, respectively. Additionally, the pH sensitivity of the conducting films was tested, PPy-b-PCaprol showed a pH-response in the interval of 4–9, and Meth-PEDOT in the pH interval 2–4. After characterizing the conducting materials, they were used as a solid contact to fabricate a pH-sensitive SCISE with the photocured polyurethane ion-selective membrane developed earlier. All pH-SCISEs showed close to Nernstian response; nevertheless, pH-SCISEs based on PEDOT:PEG exhibited better potentiometric response in terms of sensitivity, reproducibility, and lifetime. Obtained experimental data suggest that commercial PEDOT:PEG is the best candidate to be used for mass production of single-used SCISEs. The PEDOT:PEG sensor was used to measure pH of different real samples showing satisfactory results. 

## Figures and Tables

**Table 1:** Results of the chronopotentiometry measurements where *R*, Δ*E*/Δt and *C*_L_ are the bulk resistance of the ISM, potential drift of the pH-SCISEs and the redox capacitance of the solid contact.

Solid-Contact	R(MΩ)	ΔE/Δt (V/s)	C_L_ (µF)
Pure gold electrode	26.1	1.1 × 10^−3^	0.8
PPy-PCaprolac	81.3	2.5 × 10^−5^	39.2
Meth-PEDOT	61.8	3.0·× 10^−4^	3.3
Graphene/PEDOT:PSS	60.3	3.8·× 10^−5^	26
PEI + PEDOT:PSS	62	3.0·× 10^−4^	3.3
PEDOT:PSS high conductivity	57.3	2.2·10^−5^	44.8
PEDOT:PEG	65	1.8·10^−5^	54.6

**Table 2:** Parameters of the studied pH-SCISEs: potentiometric slopes (S) of the linear part of the calibration curves, standard potentials (*E*^0^) and their reproducibility (*n* = 3).

Solid-Contact	S (mV/pH)	E^0^ (mV)	Linear Range (pH)
PPy-bPCaprol	52.3 ± 0.12	420.6 ± 8.1	2–7
Graphene/PEDOT:PSS ink	54.5 ± 7.4	365.1 ± 98.8	3.5–7
Meth-PEDOT	53.0 ± 1.7	268.0 ± 19.4	3–7
PEI + PEDOT:PSS	49.4 ± 2.0	163.2 ± 38.5	2–8.5
PEDOT:PEG	53.9 ± 1.2	308.5 ± 37.2	3–8
PEDOT:PSS high conductivity	51.3 ± 7.4	326.2 ± 211.4	3–7

**Table 3:** The results of the pH determination in different samples (standard deviations were calculated for three different electrodes and five consecutive measurements).

Sample	pH _(glass pH electrode)_	pH _(pH-SCISEs based on PEDOT:PEG)_
Blood Gas Control	7.7	7.7 ± 0.15
Fish Farm	7.0	6.8 ± 0.14
Sauna	4.5	4.5 ± 0.08

## Supplementary Materials

The following are available online at https://www.mdpi.com/1424-8220/20/5/1348/s1. Figure S1: Optical images of: a) Bare electrode and b) PEDOT:PEG modified electrode. Table S1: Protocol of preparation and deposition the conducting materials.
