# Supplementary material for: Comparison of Different Commercial Conducting Materials as Ion-to-Electron Transducer Layers in Low-Cost Selective Solid-Contact Electrodes
Source: Sensors (Basel). 2020 Feb 29;20(5):1348. doi: 10.3390/s20051348 (PMC7085546; doi:10.3390/s20051348)
Supplement: Supplementary file 1 [file sensors-20-01348-s001.pdf]

Supplementary Information

The supporting information contains tables to give more information and details about the work.

Table S1. Protocol to prepare and deposit the conducting materials.

Polymer/ink	Agitation (min)	Sonication (min)	Films*1	T (ºC)*2
**Graphene/PEDOT:PSS**	*35*	-	*3*	*90*
**PPy-PCaprol**	*30*	*60*	*2*	*35*
**Meth-PEDOT**	*20*	*30*	*3*	*35*
**PEDOT:PEG**	*30*	*30*	*2*	*45*
**PEDOT:PSS hc**	*20*	-	*2*	*95*

*1 Each film correspond to one drop of 2.5 µL of the polymer material.

*2 Temperature to increase the evaporation rate of the solvent.

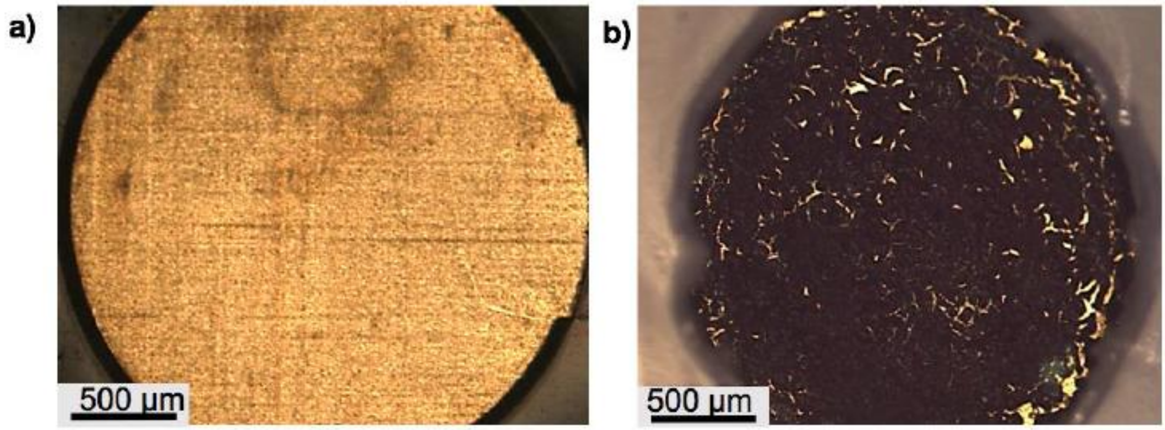

Figure S1. Optical images of: a) Bare electrode and b) PEDOT:PEG modified electrode.
